# Microplastics: a potential threat to gut microbiota and antioxidant capacity of broiler chickens

**DOI:** 10.3389/fmicb.2026.1708036

**Published:** 2026-02-17

**Authors:** Xinxi Qin, Shuai Song, Guoqing Xiang, Shengjun Luo, Xiaohui Wen

**Affiliations:** 1 Institute of Animal Health, Guangdong Academy of Agricultural Sciences, Guangzhou, China; 2 Key Laboratory for Prevention and Control of Avian Influenza and Other Major Poultry Diseases, Ministry of Agriculture and Rural Affairs, Guangzhou, China; 3 Guangdong Province Key Laboratory of Livestock Disease Prevention, Guangzhou, China; 4 Department of Animal Science, College of Biology and Food, Shangqiu Normal University, Shangqiu, China

**Keywords:** antioxidant, biochemical, chicken, gut microbiota, microplastics

## Abstract

The detrimental effects of microplastics on environmental and biological health have been extensively documented, encompassing various aspects such as growth inhibition, metabolic disorders, and organ damage. However, current research predominantly focuses on model organisms, with limited studies investigating their effects on broiler chickens. Therefore, this study aims to examine the impact of microplastics exposure on the gut microbiota and antioxidant function in broiler chickens. The results indicated that microplastics significantly affect serum biochemical and antioxidant parameters, evidenced by marked increases in AST, ALT, and MDA levels, alongside decreases in SOD and GSH-Px levels. Microbiome analysis revealed a significant decrease in the alpha diversity of the gut microbiota, accompanied by significant alterations in microbial structure. Additionally, metastats analysis demonstrated a significant increase in the relative abundances of one phylum and 12 genera during microplastics exposure, contrasted with a significant decrease in the relative abundances of three phyla and 108 genera. Importantly, microplastics exposure also led to changes in gut microbial function, affecting energy metabolism, coenzyme transport and metabolism, and amino acid metabolism, etc. In summary, our study demonstrates that microplastics can adversely affect the health of broiler chickens by reducing their antioxidant capacity, and causing gut microbial dysbiosis. In light of the increasing pollution from microplastics, this study provides crucial information for assessing the risks posed by microplastics to livestock production. Furthermore, future research should prioritize monitoring the migration of microplastics within the food chain and examining their long-term effects on biological behavior and ecological functions.

## Introduction

Plastic products, including disposable tableware, packaging materials, automobiles, and agricultural items, have experienced substantial growth, significantly enhancing the convenience of daily life. According to statistics, global plastic production has increased from 2 million tons per year in 1950 to 400 million tons per year in 2022, and is expected to reach 800 million tons by 2050 (Okoffo et al., 2021; Balabantaray et al., 2023). China ranks among the leading producers and consumers of plastics, with an annual consumption exceeding 80 million tons. However, most plastic products cannot be effectively recycled, but are disposed of by deep burial and incineration (Zimmermann et al., 2021). Notably, these plastic wastes can degrade into microplastics via biodegradation, photodegradation, and ultraviolet irradiation (Beer et al., 2018). Microplastics are defined as plastic fragments and particles with a diameter of less than 5 mm, representing an emerging pollutant that has garnered significant attention (Luo et al., 2022). Oceans, lakes, and terrestrial environments serve as the primary repositories for microplastics, indicating that organisms inhabiting these ecosystems are inevitably affected (Shaw et al., 2023; Gambino et al., 2025). Furthermore, the presence of microplastics has been detected in various plants, seafood, and vegetables, suggesting that both animals and humans may ingest microplastics through the food chain (Rillig et al., 2019). Numerous studies have indicated the detrimental effects of microplastics on host health. For instance, Liu et al. demonstrated that exposure to microplastics can induce liver lipid metabolism disorders in mice, accompanied by an gut microbial imbalance (Liu et al., 2023). Similarly, Yang et al. (2022) found that microplastics negatively impact mice fetuses by inducing oxidative damage and inhibiting GABA synthesis in the fetal brain. However, current research on microplastics predominantly focuses on model organisms, with limited studies on farmed animals. Previous studies have demonstrated that microplastics can transfer from soil to earthworms and subsequently to chickens under field conditions, indicating a potential impact on chicken health (Papazlatani et al., 2024). Additionally, Wu et al. (2021) identified the presence of microplastics in livestock feces in South China, suggesting possible exposure in poultry farming. Consequently, it is imperative to explore the detrimental effects of microplastics exposure on chicken health. In broilers, dose/form-dependent microplastics effects on antioxidant systems and cecal microbiota remain undefined. Consequently, it is necessary to explore the detrimental effects of microplastics exposure on poultry health.

The gut microbiota is a symbiotic microbial community residing in the host's intestine, comprising over 100 trillion microorganisms, including bacteria, fungi, and viruses (Li Y. et al., 2025; Ren et al., 2023). This community plays a crucial role in maintaining the host's health by establishing a symbiotic relationship with the host (Hou et al., 2025). Research has demonstrated that the gut microbiota is essential for intestinal digestion, absorption, metabolism, and the integrity of the intestinal barrier (Tong et al., 2022). Moreover, some studies on the gut microbiota have also revealed its important contributions to extraintestinal systems, such as bone development, immune system maturation, and resistance to pathogenic bacterial invasion (Chang et al., 2025; Guo, 2021). The stability of the gut microbiota is vital for the maintenance of host health and various complex physiological functions. However, this stability can be easily disrupted by a range of external and internal factors. Gender and age are primary internal factors influencing the composition of the gut microbiota, typically resulting in minimal changes (Wang et al., 2022). In contrast, strong stimuli such as heavy metals, antibiotics, microplastics, and pesticides can induce significant alterations in the gut microbiota, leading to gut microbial dysbiosis (Bariod et al., 2025). Studies indicated that approximately 90% of diseases are closely associated with gut microbial dysbiosis. For instance, gut microbial dysbiosis may result in a series of gastrointestinal symptoms, ranging from mild cramps and diarrhea to more severe chronic diseases (Ma et al., 2020). Moreover, gut microbiota is also closely related to the development of diseases such as the immune system (allergies, autoimmune diseases), endocrine system (obesity, hyperlipidemia, diabetes), nervous system (Parkinson's disease), respiratory system (asthma, pneumonia), and mental system (depression, anxiety) (Bian et al., 2025; Nie et al., 2019; Wilkins and Reimer, 2021). Therefore, maintaining the stability of gut microbiota is essential for host health.

The output and consumption of chickens have experienced substantial growth over the past few decades, driven by population growth and the high nutritional value of chicken meat. According to statistics, global chicken consumption is projected to reach 102.266 million tons by 2025, representing a year-on-year increase of 1.67%. As one of the largest consumers, China accounts for approximately 15.3 million tons of chicken consumption annually, constituting about 15% of the global total. In the context of global poultry meat consumption, the proportion of chicken is anticipated to rise from 39% during the baseline period (2018–2020) to 41% by 2030, becoming the main source of protein. Consequently, any factor that endangers the health of chickens warrants significant attention. Research indicated that plastic products are extensively utilized in animal husbandry, including feed troughs, drinking troughs, and plastic bottles (Li et al., 2023). During this period, plastic fragments may degrade into microplastics through chemical, physical, and/or biological interactions, subsequently entering the digestive tract via the food chain and ultimately being ingested by chickens. Recent surveys have documented the presence of microplastics in poultry feces, providing evidence for the ingestion of these particles by chickens (Wu et al., 2021). However, there is a scarcity of studies examining the effects of microplastics on the gut microbiota of chickens. Thus, we hypothesized that microplastics could affect chicken health by reducing antioxidant capacity and altering cecal microbiota composition.

## Materials and methods

### Experimental design and sample acquisition

A group of 40 1-day-old AA chickens was procured from a commercial farm for the purpose of conducting animal experiments. These chickens exhibited comparable body weights and underwent a health evaluation prior to the experiment. They were housed collectively for a duration of 3 days before being randomly assigned to two distinct groups: the control group (CON) and the microplastics intake group (MIC). These chickens were randomly assigned regardless of sex, with 20 chickens per group. The rearing conditions for both groups, including temperature (33 °C~35 °C), humidity (60%−65%), and lighting (23 h/1 h light/dark cycle), was in accordance with the recommendations of the standard management manual. Furthermore, both groups were provided with an adequate diet and access to drinking water throughout the 28-day experimental period. Notably, the chickens in the microplastics intake group received feed supplemented with microplastics (300 mg/kg) to investigate the potential effects of microplastics on their health. The dosage of microplastics and the duration of the experimental period were determined based on findings from previous studies (2023). The average daily feed intake and changes in body weight of the chickens were meticulously recorded during the experiment. After the experiment, all chickens were euthanized by injecting pentobarbital (25 mg/kg) (Qin et al., 2025). Additionally, the spleen, bursa of Fabricius, thymus, and cecal contents were collected for the calculation of immune organ indices as well as for intestinal microbial and metabolomic analyses.

### Biochemical assays

Detection of the catalase (CAT), total antioxidant capacity (T-AOC), glutathion peroxidase (GSH-Px), superoxide dismutase (SOD), malonaldehyde (MDA), ALT (alanine transaminase) and AST (glutamic oxalacetic transaminase) levels were conducted in accordance with the recommendations of the commercial kits (Nanjing Jiancheng Bioengineering Institute, Nanjing, China).

### Amplicon sequencing of gut microbiota

The detailed methods and procedures for amplicon sequencing of the gut microbiota were consistent with those described in prior studies (Li et al., 2024; Hou et al., 2025; Hu et al., 2016). In summary, DNA was extracted from the cecal content samples of broiler chickens using a commercial extraction kit (QIAGEN, H; ilden, Germany). Subsequently, the extracted DNA samples were assessed for both concentration and integrity. The V3-V4 region of the bacterial 16S rRNA gene was amplified using universal primers (338F: ACTCCTACGGGAGGCAGCA and 806R: GGACTACHVGGGTWTCTAAT). All PCR amplifications were conducted in triplicate to ensure accuracy. To differentiate each sequenced sample and obtain precise phylogenetic and taxonomic information, forward and reverse error-correction barcodes were added to the gene products. Following purification, the amplicons were quantified, and equimolar normalized amplicons were pooled. Finally, sequencing was performed on a MiSeq PE300 sequencer (Illumina, CA, USA) utilizing a 2 × 300 bp sequencing protocol in accordance with the manufacturer's instructions.

### Sequencing data analysis

Generated raw sequences were assigned to corresponding samples based on barcode information. However, these raw sequences required further processing to obtain usable, high-quality sequences due to the presence of erroneous, short, or ambiguous sequences. Sequences exhibiting over 97% similarity were subsequently categorized into specific OTUs. To evaluate the diversity and abundance of gut microbiota within the samples, both alpha and beta diversity analyses were conducted. Alpha diversity was determined based on the abundance of OTUs within each sample. Additionally, beta diversity was assessed by generating PCoA plots to visualize structural differences in gut microbiota. In this study, PICRUSt2 method was used to predict the gene family abundances of bacterial communities as per the 16S rDNA gene data and a database of reference genomes (Hu et al., 2016). Metastats analysis was employed to compare differences in bacterial abundance, while LEfSe analysis was utilized to identify biomarkers associated with microplastics exposure. Statistical analyses were performed using GraphPad Prism (v9.0), with data presented as mean ± SD. Differentially expressed bacteria and biomarkers were identified with a *P*-value < 0.05 or LDA score > 4, respectively.

## Results

### Growth parameter analysis

**Figure 1:**
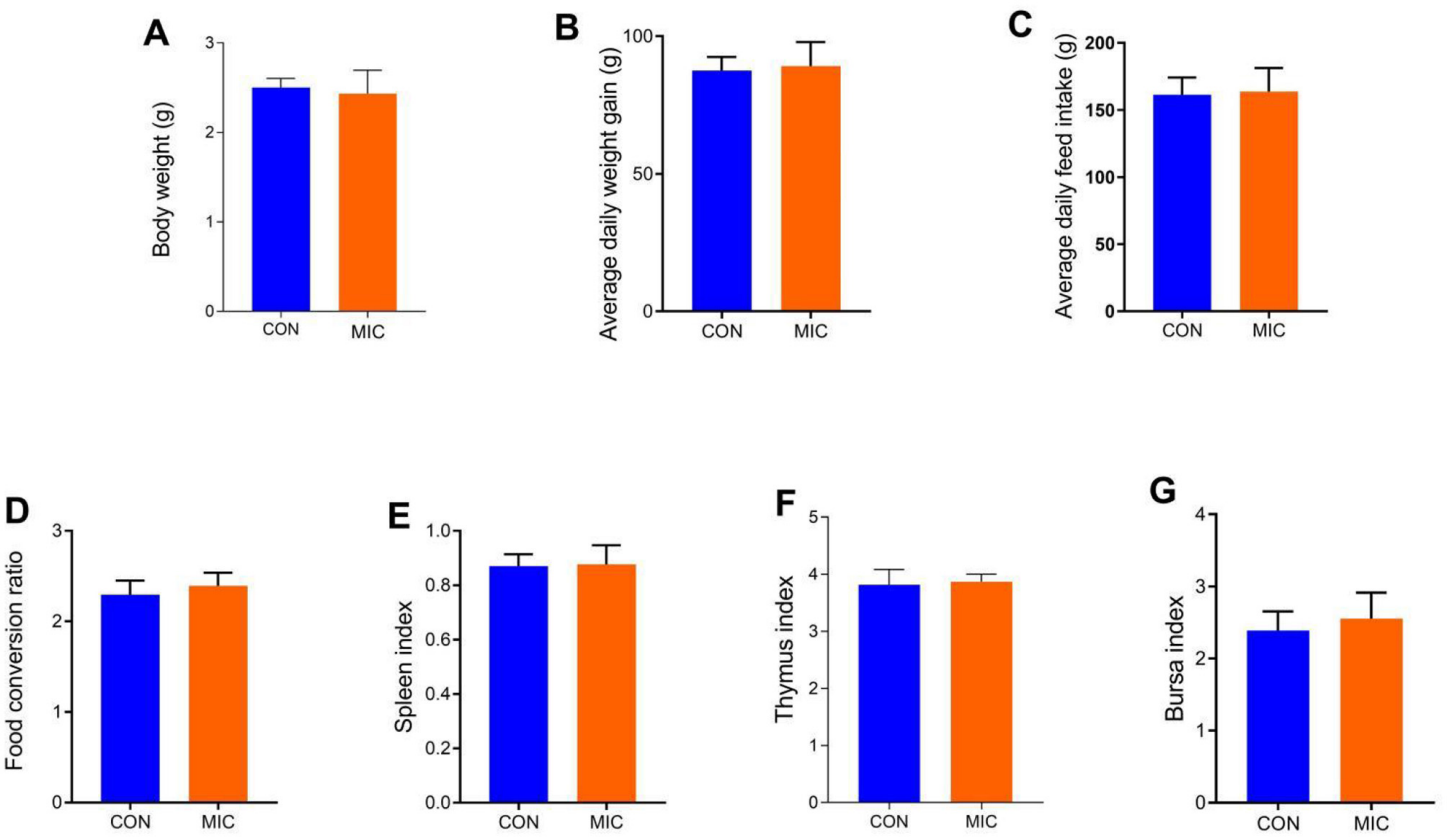
Effects of microplastics exposure on growth parameter in broiler chickens. **(A)** body weight; **(B)** average daily weight gain; **(C)** average daily feed intake; **(D)** food conversion ratio; **(E)** spleen index; **(F)** thymus index; **(G)** bursa index. Data were represented as means ± SD.

Figure 1 illustrates the changes in growth performance-related parameters of broiler chickens resulting from microplastic ingestion. The findings indicate that microplastics ingestion did not significantly affect the growth performance and organ indices of broilers (*P* > 0.05).

### Microplastics cause transaminase abnormalities and reduce host antioxidant capacity

**Figure 2:**
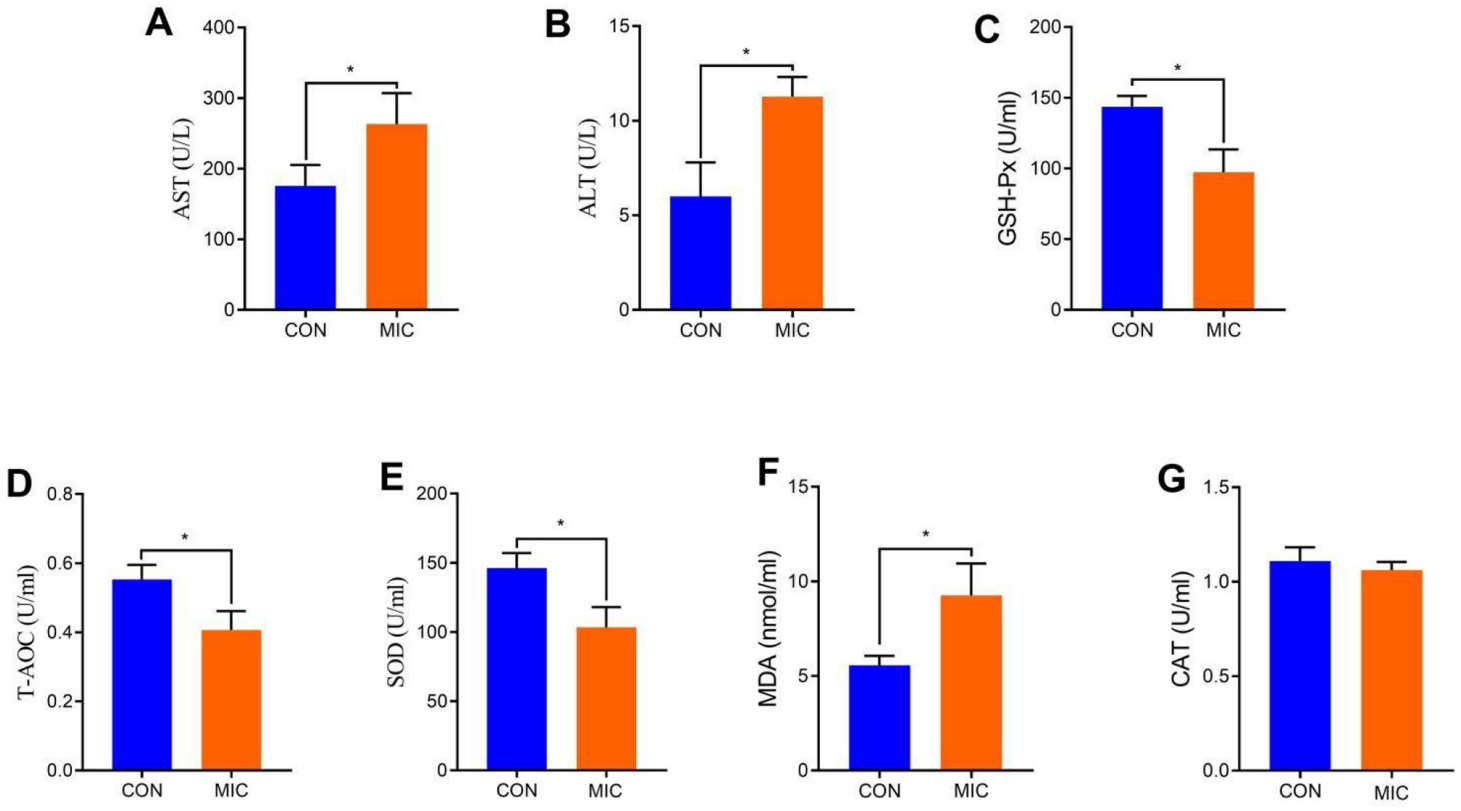
Effects of microplastics exposure on serum biochemistry, antioxidants and cytokines in broiler chickens. **(A)** AST; **(B)** ALT; **(C)** GSH-Px; **(D)** T-AOC; **(E)** SOD; **(F)** MDA; **(G)** CAT. Data were represented as means ± SD. * *P* < 0.05.

The results indicated that serum biochemical indicators, including AST and ALT, significantly increased during microplastics exposure (*P* < 0.05) (Figures 2A, B). Furthermore, microplastics exposure resulted in a notable decrease in T-AOC, GSH-Px and SOD levels, alongside a significant increase in MDA level in broiler chickens (*P* < 0.05) (Figures 2C–F). Notably, microplastics exposure did not affect serum CAT level (*P* > 0.05) (Figure 2G).

### Sequence analysis

**Figure 3:**
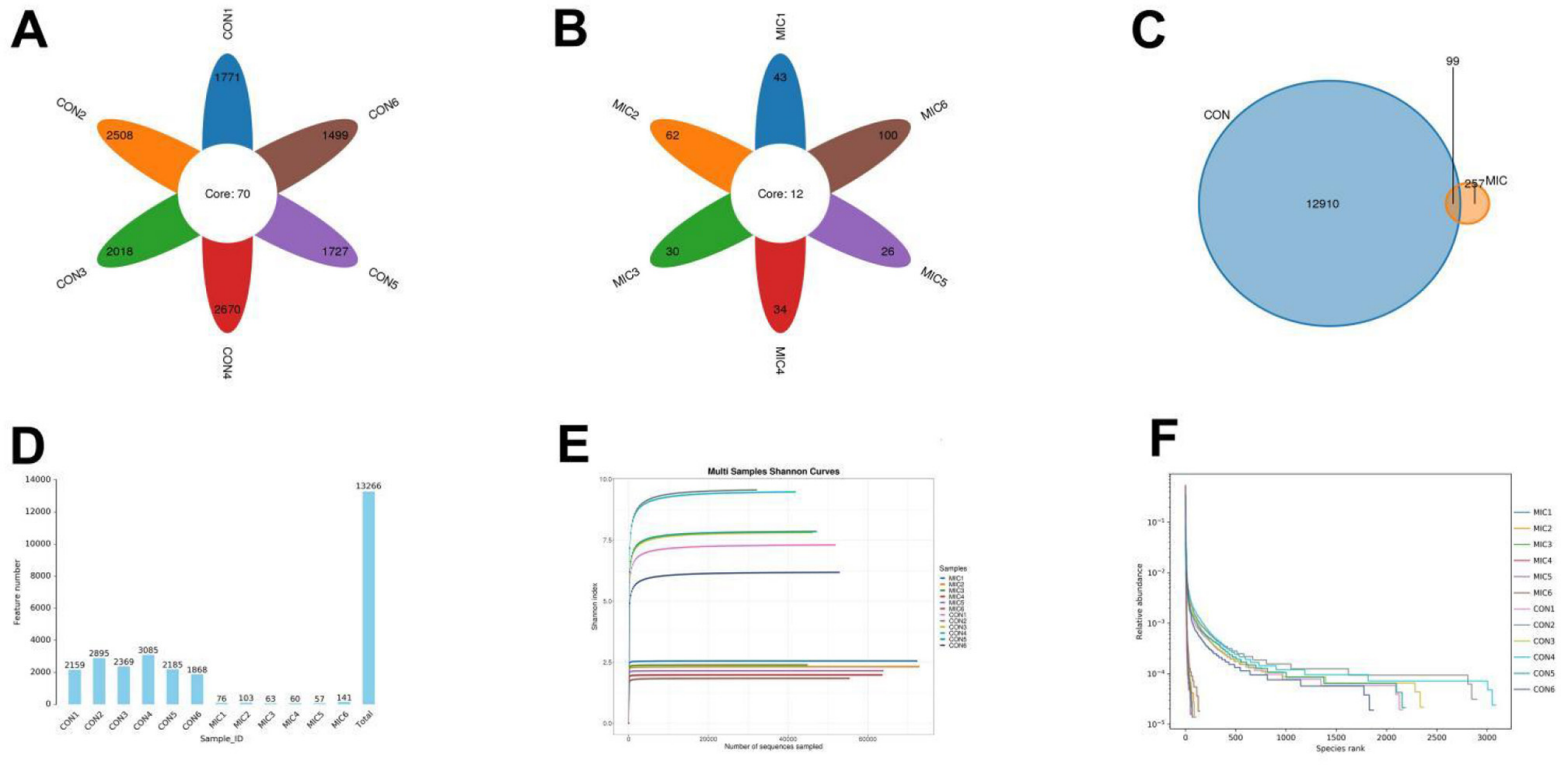
Rarefaction curve and OTUs cluster analysis. **(A, B, C)** Venn diagram was generated based on the valid sequences of various sample species. This map provides a visual representation of the distribution of OTUs within the samples analyzed. **(D, E, F)** Rarefaction curves for evaluating sequencing depth.

In this study, amplicon sequencing generated a total of 1,360,105 (CON = 777,753, MIC = 582,352) raw sequences, averaging 113,342 sequences per sample. After filtering out unqualified sequences, we obtained a total of 673,697 (CON = 299,676, MIC = 374,021) valid sequences across the two groups, resulting in a qualification rate of ~50% (Supplementary Table 1). These qualified sequences were subsequently clustered into 13,266 OTUs, with 13,009 OTUs in the CON and 356 OTUs in the MIC (Figures 3A–D). Among these identified OTUs, 99 OTUs appeared in both the CON and MIC. Additionally, the number of unique OTUs was 12,910 in the CON and 257 in the MIC. We also constructed the rarefaction curve to evaluate sequencing depth, which indicated that all curves exhibited a saturation trend, demonstrating the adequate sequencing depth (Figures 3E, F).

### Effects of microplastics exposure on gut microbial diversity

**Figure 4:**
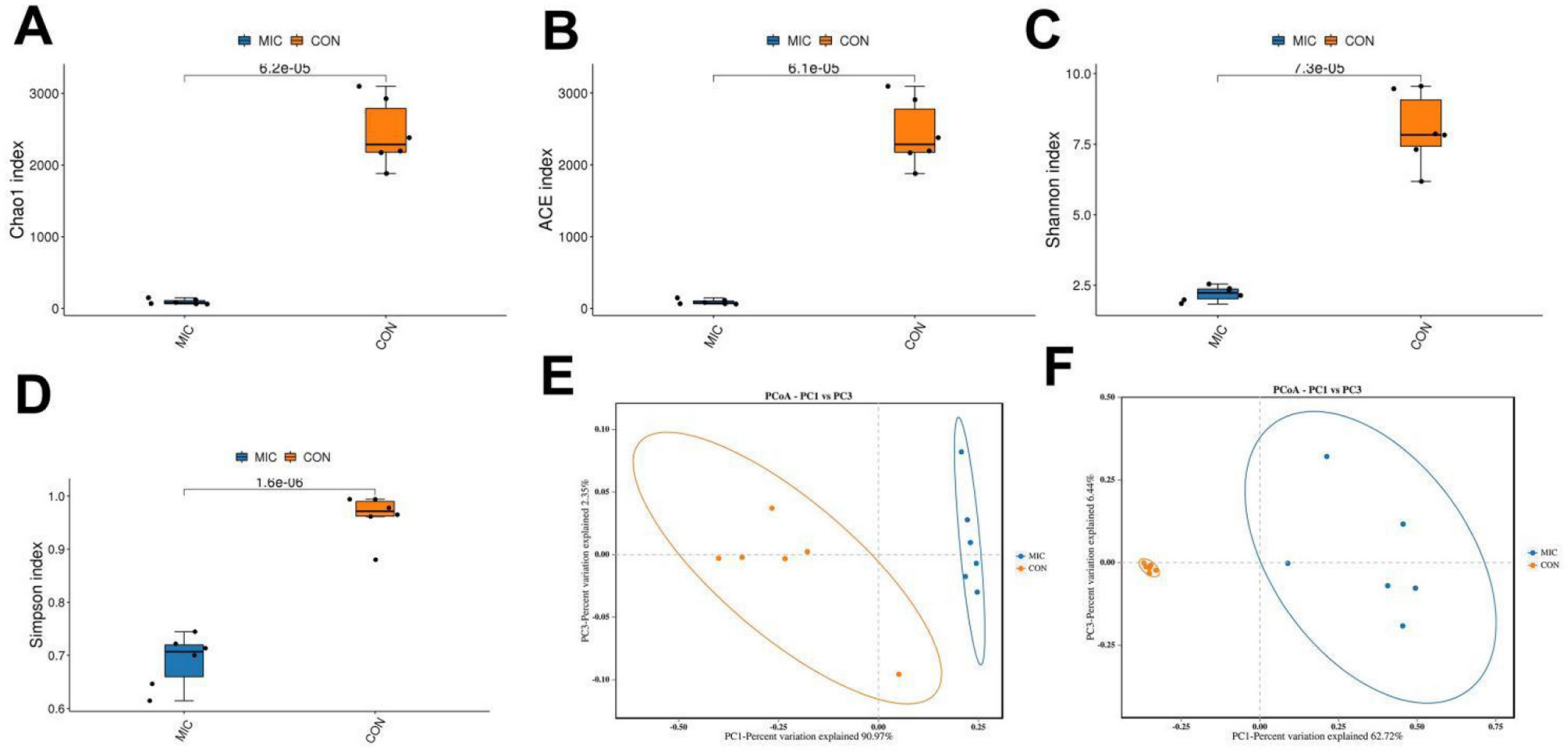
Microplastics exposure altered the gut microbiota diversity. Alpha diversity reflects the species richness (**A**: ACE, **B**: Chao1) and species diversity (**C**: Shannon, **D**: Simpson). Beta diversity reflects the gut microbial structure (**E, F**: PCoA scatter plots).

The alpha diversity index was computed according to the number of OTUs in each sample group to assess the impact of microplastics on gut microbial diversity. The results indicated that the average values of the Chao1, ACE, Shannon, and Simpson indices in the CON were 2,441.62, 2,435.69, 8.02 and 0.96, respectively. Furthermore, the corresponding values in the MIC were 89.93, 87.75, 2.20 and 0.69, respectively. Comparative analysis of microbial diversity revealed significant differences in the Chao1 (2,441.62 ± 192.90 vs. 89.93 ± 14.38, *P* < 0.0001), ACE (2,435.69 ± 191.64 vs. 87.75 ± 13.93, *P* < 0.0001), Simpson (0.96 ± 0.017 vs. 0.69 ± 0.020, *P* < 0.0001), and Shannon (8.02 ± 0.53 vs. 2.20 ± 0.10, *P* < 0.0001) indices between the two groups, suggesting that microplastics dramatically decreased the diversity and abundance of the gut microbiota in chickens (Figures 4A–D). Furthermore, we constructed PCoA scatter plots for beta diversity analysis. The beta diversity analysis demonstrated that the samples from both the CON and MIC did not cluster together, indicating that the structure of the gut microbiota was significantly affected by microplastics (Figures 4E, F).

### Effects of microplastics exposure on gut microbial composition

**Figure 5:**
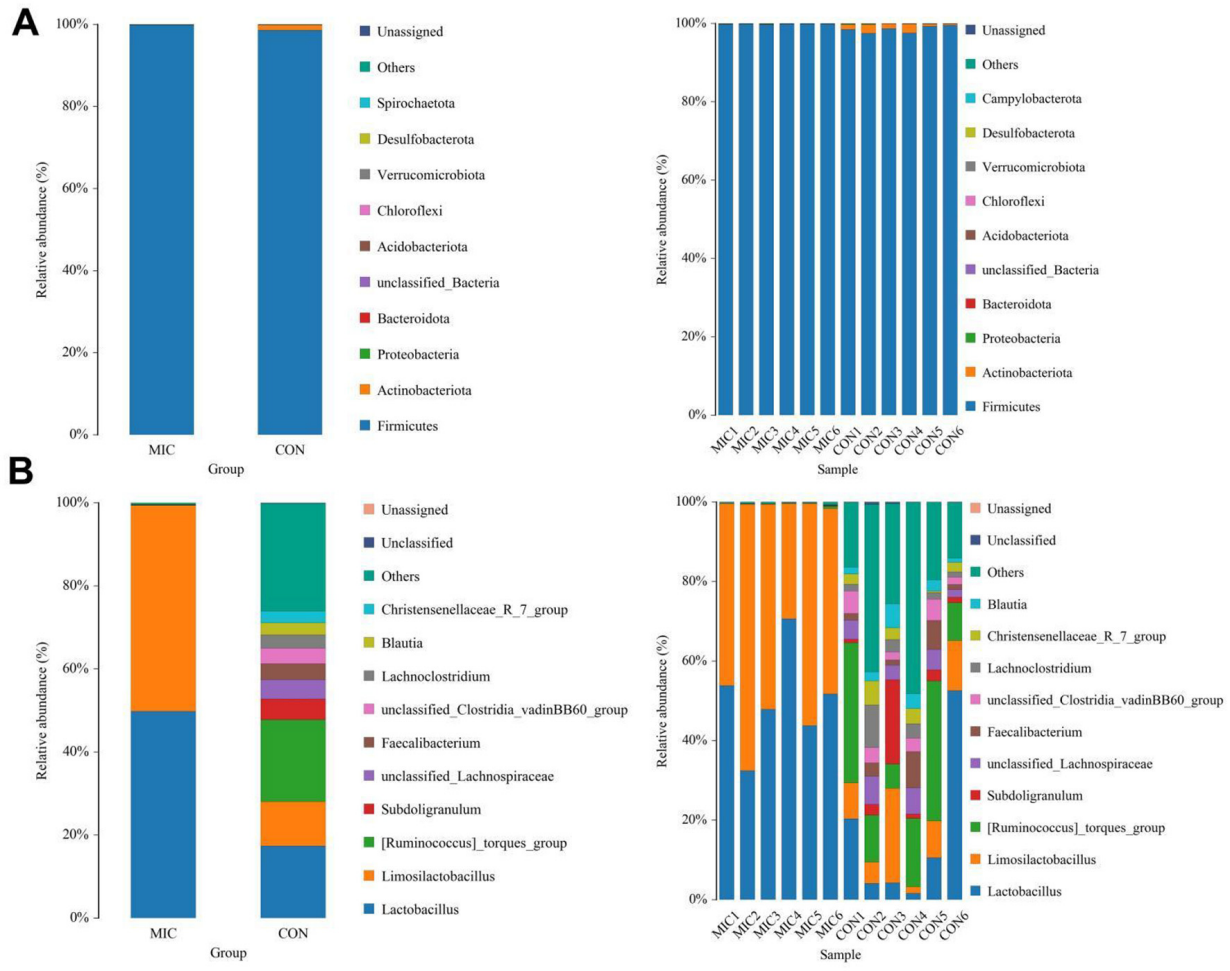
Effects of microplastics exposure on gut microbiota compositions at phylum **(A)** and genus **(B)** levels in broiler chickens. The length of each bar corresponds to the relative abundance ratio of the respective species, providing a clear visual representation of species diversity within the sample.

**Figure 6:**
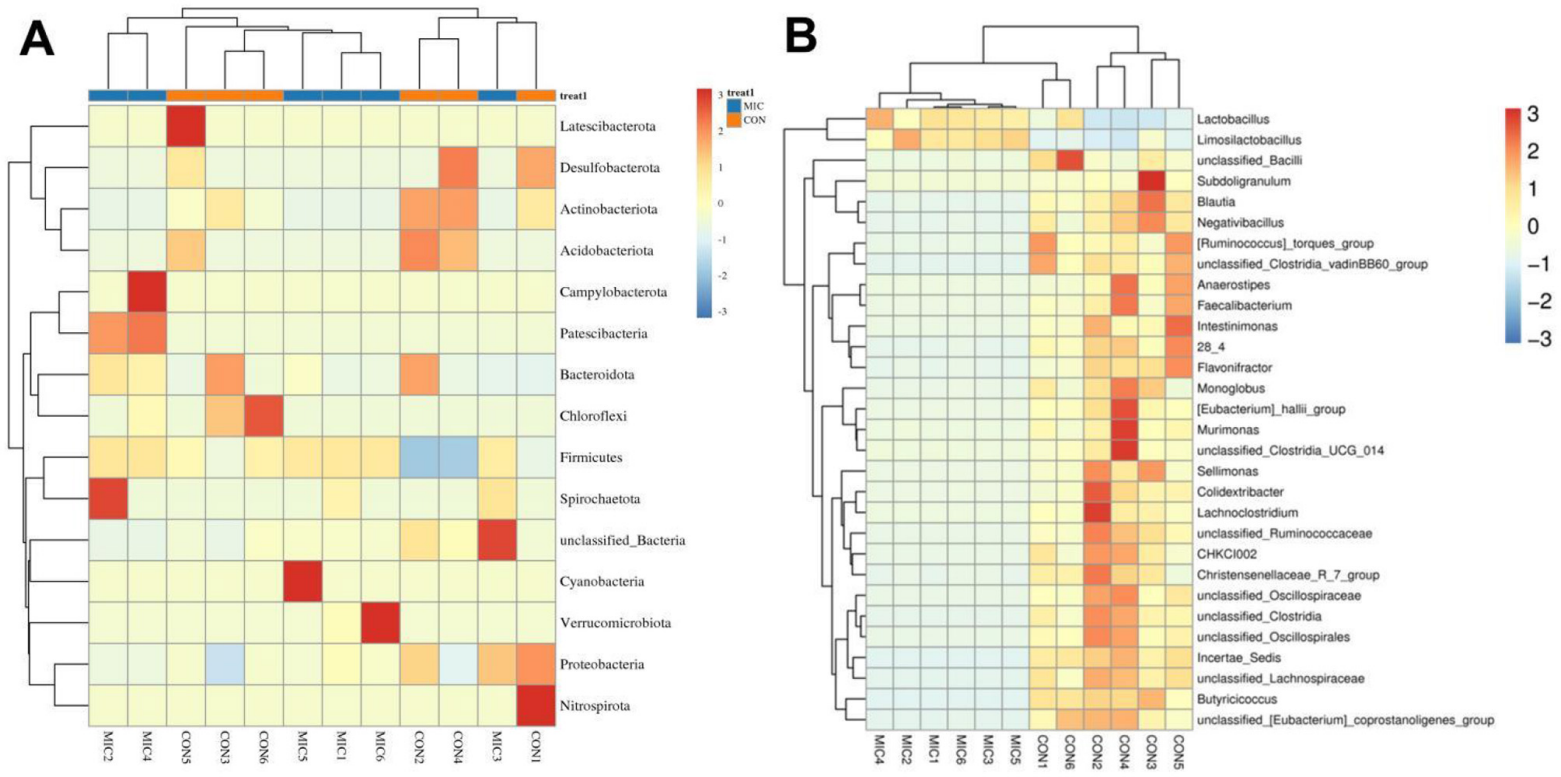
Heat map and hierarchical clustering of changed phyla **(A)** and bacteria **(B)** during microplastics exposure. The similarities and differences in community composition across multiple samples are represented by color gradients and levels of similarity.

Figure 5 illustrates the ten most abundant bacterial phyla and genera of the gut microbiota. More precisely, the phyla *Firmicutes* (98.61%, 99.84%), *Actinobacteriota* (1.21%, 0.016%), *Proteobacteria* (0.098%, 0.089%) and *Bacteroidota* (0.025%, 0.023%) were the four most dominant bacteria in CON and MIC (Figure 5A). Other bacterial phyla including *Acidobacteriota, Chloroflexi, Verrucomicrobiota, Desulfobacterota* and *Spirochaetota* were identified with abundances less than 0.001%. At the genus level, *[Ruminococcus]_torques_group* (19.76%) the most prevalent bacteria in the CON followed by the *Lactobacillus* (17.38%) and *Limosilactobacillus* (10.65%). Furthermore, *Lactobacillus* (49.77%), *Limosilactobacillus* (49.62%) and *[Ruminococcus]_torques_group* (0.10%) were abundantly present in the MIC (Figure 5B). Notably, we also generated heat maps to further illustrate the abundance and distribution of more bacterial phyla and genera (Figure 6).

**Figure 7:**
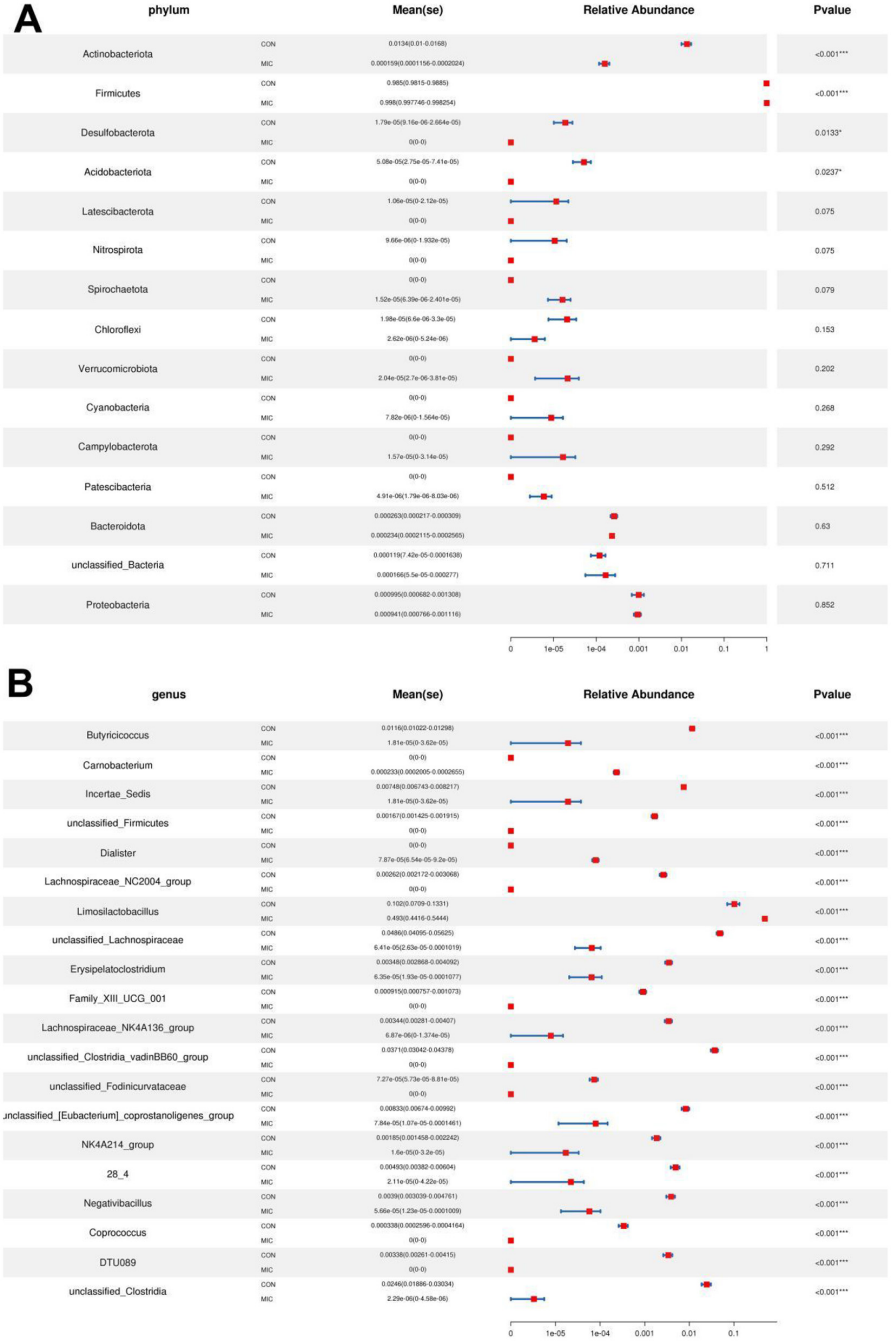
Statistical analysis of bacteria with significant differences between the CON and MIC. **(A, B)** Changes in relative abundance at the phylum and genus levels. *p < 0.05, ***p < 0.001.

**Figure 8:**
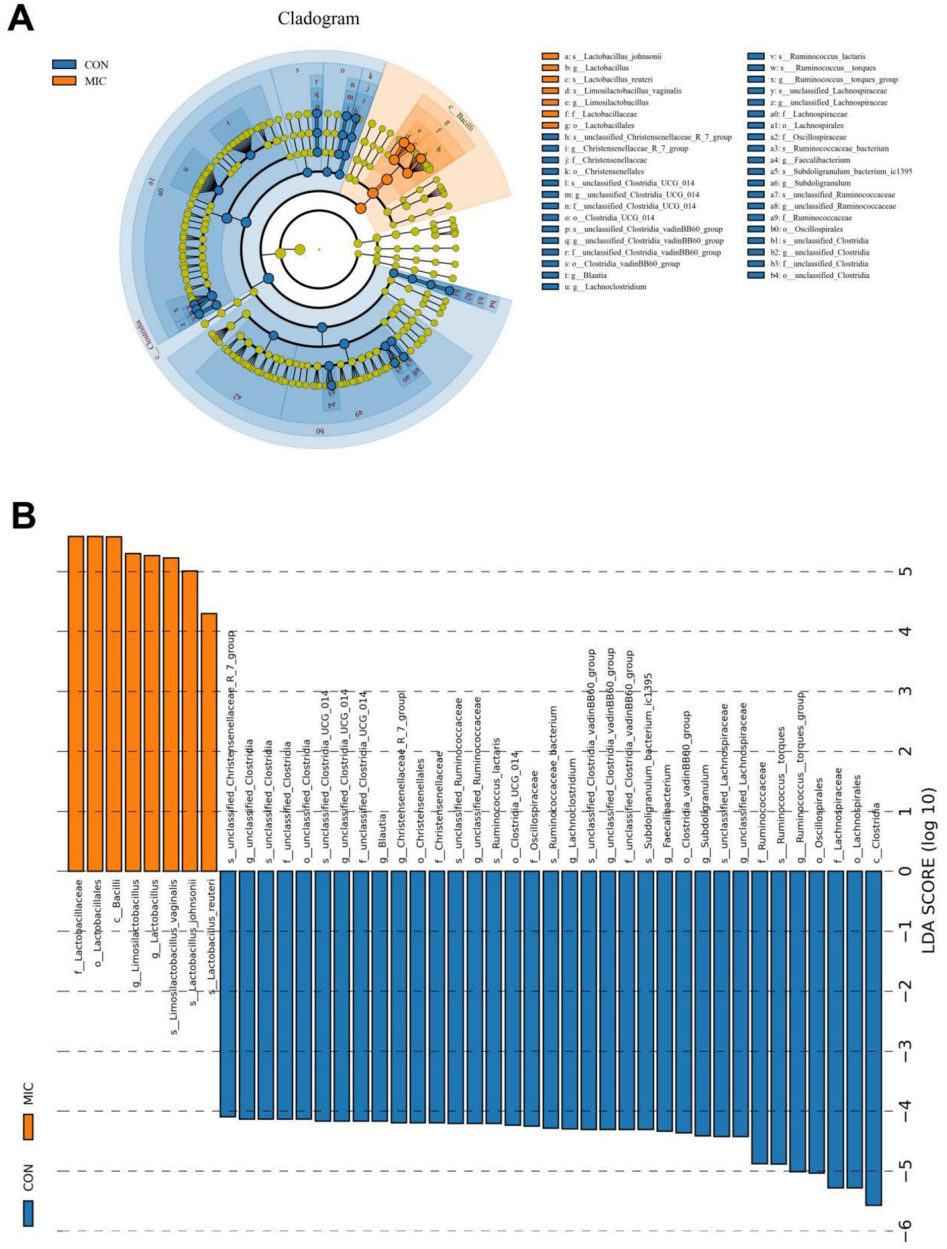
Biomarkers with statistically significant differences between the CON and MIC. **(A)** Cladogram of species evolution. The circles radiating outward from the center of the cladogram represent the classification levels, ranging from phylum to species. **(B)** Distribution histogram of LDA values of differential biomarkers.

Metastats analysis indicated that the abundances of *Actinobacteriota, Desulfobacterota* and *Acidobacteriota* decreased significantly, while the abundance of *Firmicutes* increased significantly during microplastics exposure (Figure 7A). Moreover, we also found that 120 genera were significantly different between the CON and MIC (Figure 7B). Among them, the levels of 12 bacterial genera (*Carnobacterium, Dialister, Limosilactobacillus, Klebsiella, Lactobacillus, Phascolarctobacterium, Kroppenstedtia, Weissella, Sphingomonas, Collinsella, Paucibacter, Staphylococcus*) significantly increased, whereas the levels of 108 bacterial genera (*Butyricicoccus, Lachnospiraceae_NC2004_group, Erysipelatoclostridium, Lachnospiraceae_NK4A136_group, Negativibacillus, Coprococcus, unclassified_Clostridia, Blautia, Gordonibacter, Lactonifactor, Flavonifractor, Christensenellaceae_R_7_group, Sporobacter, [Ruminococcus]_torques_group, Anaerofustis, Bacillus, Oscillibacter, Monoglobus, Roseburia, Caproiciproducens, Ileibacterium, Ruminococcus, Intestinimonas, Sellimonas, Pontibacter, Ligilactobacillus, Faecalibacterium, Colidextribacter, Lactococcus, Fournierella, Frisingicoccus, Anaerotruncus, Shuttleworthia, Lachnospiraceae_ND3007_group, Lachnoclostridium, Marvinbryantia, Anaerostipes, Lachnospiraceae_UCG_008, Papillibacter, Peptococcus, Thermobifida, Paludicola, Murimonas, Catenibacillus, Enterorhabdus, Anaerocolumna, Anaerosporobacter, Anaerostignum, Angelakisella, Anseongella, Bryobacter, Dysgonomonas, Lachnospiraceae_UCG_004, Lachnospiraceae_AC2044_group, Lachnospiraceae_UCG_010, Lachnospiraceae_FCS020_group, Bifidobacterium*, etc.) significantly decreased during microplastics exposure. More importantly, microplastics exposure even caused 11 bacterial genera to completely disappear from the gut microbiota. Additionally, LEfSe analysis was applied to further identify the differential bacteria (Figure 8).

### Correlation network analysis

**Figure 9:**
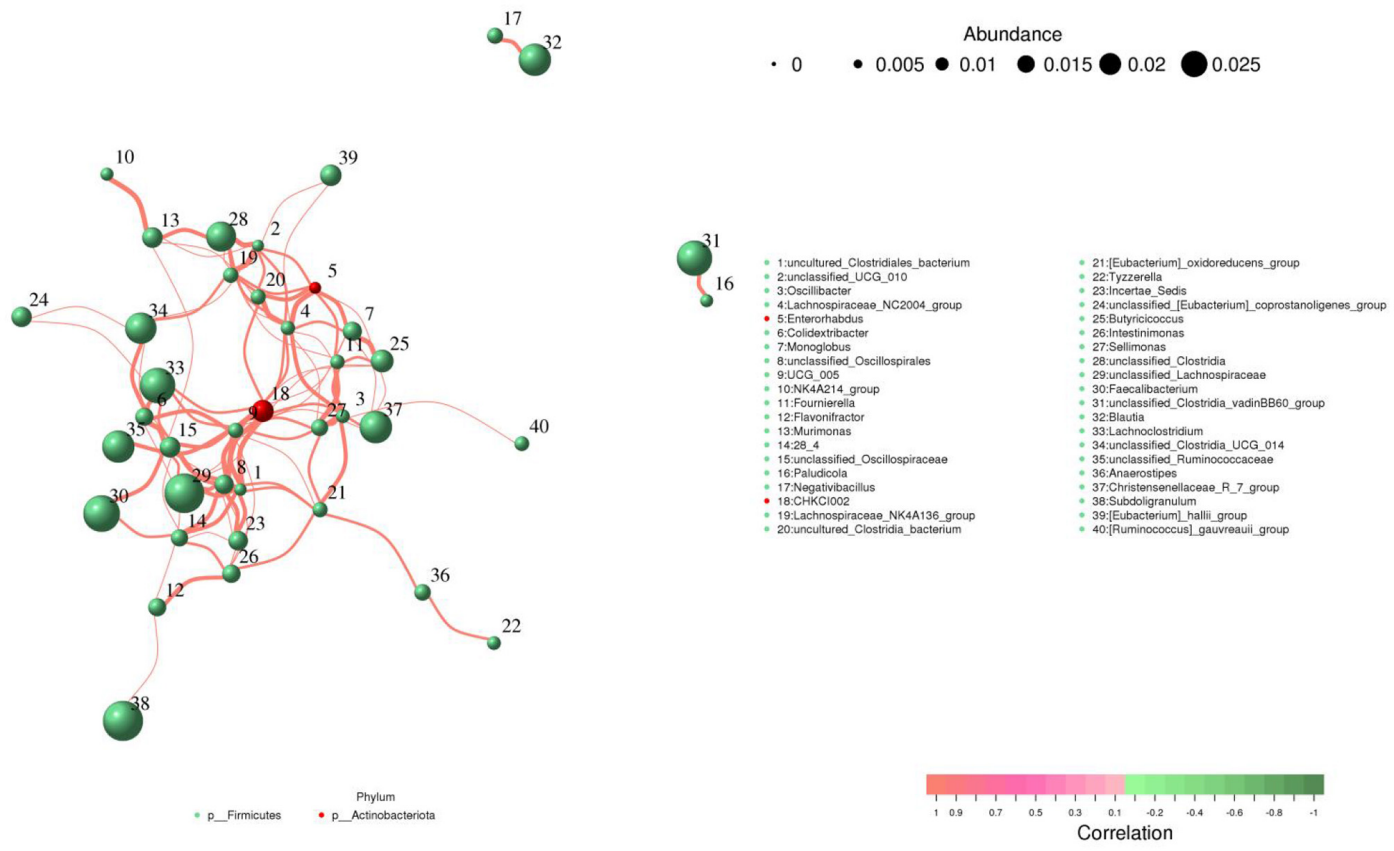
Comprehensive correlation network analysis among representative bacteria. Bacterial species and their abundance are represented by circles of varying colors, while direct correlations between bacterial species are illustrated through connecting lines.

*Lachnospiraceae_NC2004_group* was positively associated with *uncultured_Clostridia_bacterium* (0.99), *unclassified_Clostridia* (0.95), *CHKCI002* (0.95), *Monoglobus* (0.95), *UCG_005* (0.95), *[Eubacterium]_hallii_group* (0.95), *Sellimonas* (0.95), *Fournierella* (0.95), and *Lachnospiraceae_NK4A136_group* (0.94) (Figure 9). *Monoglobus* was positively associated with *Butyricicoccus* (0.97), *Enterorhabdus* (0.97), *CHKCI002* (0.95), *UCG_005* (0.95) and *Christensenellaceae_R_7_group* (0.94). *Enterorhabdus* was positively associated with *Lachnospiraceae_NC2004_group* (0.98), *Monoglobus* (0.97), *uncultured_Clostridia_bacterium* (0.96), *unclassified_UCG_010* (0.96), *Fournierella* (0.96), *Lachnospiraceae_NK4A136_group* (0.95) and *Butyricicoccus* (0.94). *Oscillibacter* was positively associated with *Sellimonas* (0.99), *[Eubacterium]_oxidoreducens_group* (0.96), *Fournierella* (0.96), *Lachnospiraceae_NC2004_group* (0.96), *UCG_005* (0.96) and *[Ruminococcus]_gauvreauii_group* (0.94).

### Functional prediction analysis

**Figure 10:**
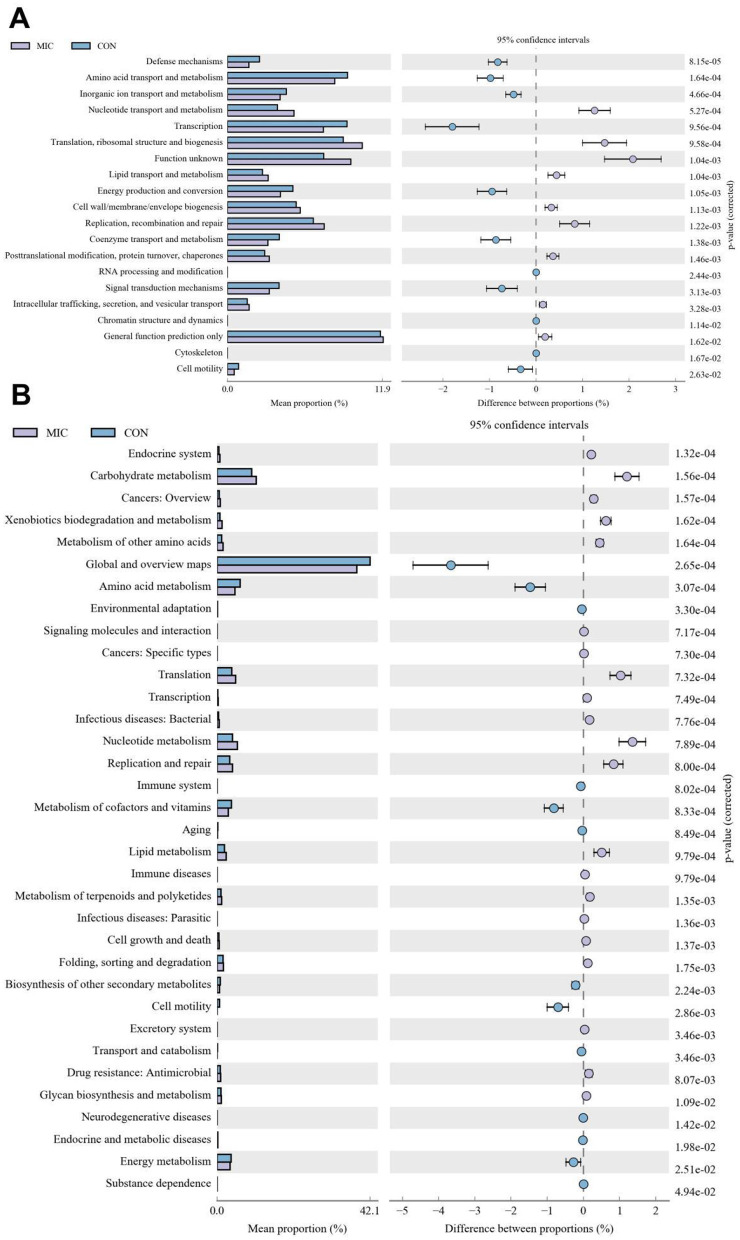
Changes in intestinal function of broiler chickens during microplastics exposure. **(A)** COG functional prediction; **(B)** KEGG function prediction.

The COG functional prediction indicated that the CON had significantly higher proportions of amino acid transport and metabolism, coenzyme transport and metabolism, transcription, cell motility, inorganic ion transport and metabolism, signal transduction mechanisms, defense mechanisms and cytoskeleton, whereas RNA processing and modification, chromatin structure and dynamics, energy production and conversion, nucleotide transport and metabolism, lipid transport and metabolism, translation, ribosomal structure and biogenesis, replication, recombination and repair, cell wall/membrane/envelope biogenesis, posttranslational modification, protein turnover, chaperones, general function prediction only and intracellular trafficking, secretion, and vesicular transport were enriched in MIC (Figure 10A). Moreover, the KEGG functional prediction indicated that the proportions of metabolism of cofactors and vitamins, energy metabolism, amino acid metabolism, biosynthesis of other secondary metabolites, global and overview maps, cell motility, transport and catabolism, aging, immune system, environmental adaptation, endocrine and metabolic diseases, neurodegenerative diseases and substance dependence in the CON was dramatically higher than that in the MIC, whereas the relative proportions of carbohydrate metabolism, lipid metabolism, nucleotide metabolism, metabolism of terpenoids and polyketides, xenobiotics biodegradation and metabolism, metabolism of other amino acids, glycan biosynthesis and metabolism, translation, drug resistance: antimicrobial, folding, sorting and degradation, transcription, replication and repair, endocrine system, signaling molecules and interaction, cell growth and death, excretory system and immune diseases was lower (Figure 10B).

## Discussion

Plastics play a crucial role in various sectors, including tableware, packaging, automobiles, and toys, thereby significantly influencing social development and human life (Li F. et al., 2025). However, the widespread use and popularity of plastic products have also triggered a new wave of ecological and environmental challenges. Surveys indicated that plastic products in the environment can degrade into microplastics through various processes, accumulating in both aquatic and terrestrial ecosystems (Hasan et al., 2024). Furthermore, microplastics can be transferred to animals, plants, and humans via the food chain, resulting in serious food safety and public health concerns (Prata et al., 2022). Numerous studies have demonstrated the detrimental effects of microplastics on water and soil quality, as well as on the health of laboratory mice (Lee et al., 2023; Chen et al., 2024). The negative impact of microplastic pollution on livestock production, particularly in poultry industry, has garnered increasing attention (Abd et al., 2025). As an important source of protein intake for residents, the health of chickens is closely related to the stability of gut microbiota. However, few studies have explored the effects of microplastics on the intestinal microbiota in chickens. This study indicated that microplastics can induce gut microbial dysbiosis.

Gut microbial diversity refers to the richness of species, their abundance, and the functional distribution of microorganisms within the intestine (Zaman et al., 2022). Research indicates that gut microbial diversity serves as a crucial indicator of the stability and functionality of the intestinal microbiome, exhibiting multidimensional associations with host health (Dong et al., 2021). The healthy gut microbiota is characterized by high diversity, which promotes nutrient metabolism, maintains immune balance, protects intestinal barrier function, and influences systemic functions through the gut-X axis (Annavajhala et al., 2020; Jabeen et al., 2023). Conversely, abnormal gut microbial diversity can contribute to the development of various diseases. For instance, individuals with obesity and diabetes exhibit significantly lower gut microbial diversity compared to healthy controls, as evidenced by reductions in the alpha diversity index and abnormal abundances of core bacterial genera (Bielka et al., 2022). Moreover, certain intestinal diseases, such as diarrhea and colitis, are also linked to significant declines in gut microbial diversity (Cheng H. et al., 2023). Previous studies have demonstrated that reduced gut microbial diversity can lead to microbial imbalances and disrupt intestinal barrier function, resulting in the overgrowth of opportunistic pathogens, the release of endotoxins into the bloodstream, and the activation of systemic inflammatory pathways (Yang R. et al., 2023). Additionally, individuals with lower gut microbial diversity tend to have elevated levels of inflammatory factors and a markedly increased risk of systemic inflammation (Xie et al., 2025). Prior research has reported that exposure to microplastics can induce gut microbial imbalances and abnormal expression of inflammatory factors (Wei et al., 2025). Consistent with these findings, we observed that microplastics exposure resulted in decreased gut microbial diversity and significantly elevated serum inflammatory cytokines in broiler chickens.

Serum antioxidant markers serve as crucial indicators for evaluating oxidative stress status and the capacity of antioxidant defense mechanisms (Akpinar et al., 2023; Lee et al., 2025). The complex bidirectional regulatory relationship exists between gut microbial dysbiosis and oxidative damage, significantly influencing the development and progression of various chronic diseases (Xu et al., 2023). Research has demonstrated that some beneficial gut bacteria can activate antioxidant enzyme systems by upregulating the activity of endogenous antioxidant enzymes, such as SOD and GSH-Px in host cells, thereby neutralizing free radicals (Arias et al., 2021). Conversely, gut microbial dysbiosis can result in the overproliferation of opportunistic pathogens, increased endotoxin release, and activation of the NF-κB pathway, causing excessive production of pro-inflammatory cytokines and reactive oxygen species, which exacerbates oxidative damage (Roy et al., 2025). Oxidative stress can directly harm intestinal epithelial cells, altering the local microenvironment, affecting microbial composition, and further aggravating gut microbial dysbiosis (Sun et al., 2024). In this study, we observed that microplastics exposure can lead to a reduction in the levels of antioxidant enzymes, inflammatory responses, and gut microbial dysbiosis. Consequently, microplastics may induce inflammatory responses and host oxidative damage by diminishing gut microbial diversity. However, the specific mechanism still requires further research.

Previous research indicated that microplastics can result in significant changes in the gut microbiota, destroying intestinal homeostasis (Cheng J. et al., 2023). Notably, similar results were also observed in this study. Specifically, some bacteria, including some beneficial bacteria (*Butyricicoccus, Lachnospiraceae_NC2004_group, Lachnospiraceae_NK4A136_group, Blautia, Gordonibacter, Christensenellaceae_R_7_group, Monoglobus, Anaerofustis, Bacillus, Oscillibacter, Roseburia, Caproiciproducens, Ruminococcus, Intestinimonas, Ligilactobacillus, Faecalibacterium, Lactococcus, Bifidobacterium, Lachnospiraceae_ND3007_group, Lachnospiraceae_UCG_008, Lachnospiraceae_AC2044_group, Lachnospiraceae_UCG_010, Lachnospiraceae_FCS020_group*, etc.), were significantly reduced during microplastics exposure. Previous studies have demonstrated that *Butyricicoccus* alleviates intestinal inflammation and contributes to the treatment of colitis (Devriese et al., 2017). The *Lachnospiraceae* not only ferments complex carbohydrates to produce metabolites that are beneficial to host health but also participates in fiber and protein metabolism, thereby influencing the efficiency of dietary nutrient conversion (Abdugheni et al., 2022). The abundance of *Coprococcus* has been positively correlated with gut microbiota diversity and can serve as a biomarker for assessing gastrointestinal health (Yang Y. et al., 2023). *Blautia* produces bacteriocins that inhibit pathogen colonization, thereby helping to maintain the balance of gut microbiota (Benitez-Paez et al., 2020; Zhong et al., 2025). Recently, *Gordonibacter* has attracted considerable attention due to its unique steroid metabolism functions. For instance, *Gordonibacter* can indirectly influence immune and inflammatory responses by regulating steroid hormone levels (Garcia-Villalba et al., 2020). *Christensenellaceae* metabolizes complex polysaccharides, such as cellulose and hemicellulose, and its abundance is significantly reduced in patients with obesity and type 2 diabetes (Guan et al., 2024). Research indicates that *Anaerofustis* can ameliorate glucose and lipid metabolism disorders by regulating bile acid metabolism and short-chain fatty acids (SCFAs) production, thereby preventing or treating metabolic diseases, including obesity and diabetes. *Anaerofustis* also shows therapeutic potential in inflammatory diseases, such as ulcerative colitis and gastritis. *Bacillus* has garnered significant attention in the field of probiotics due to its remarkable environmental adaptability and unique biological properties. Previous research indicated that *Bacillus* can substantially enhance the growth performance and antioxidant capacity of broiler chickens (Zhu et al., 2024). Moreover, *Bacillus* has the ability to consume oxygen, occupy intestinal adhesion sites, and produce antimicrobial substances that inhibit the growth of pathogens (Chen et al., 2023; Elleithy et al., 2023). *Oscillibacter*, a commensal bacterium in the gut, is known for its cholesterol-degrading capabilities (Graham, 2024). Furthermore, *Oscillibacter* may lower the risk of atherosclerosis and ischemic heart disease by reducing cholesterol absorption (Graham, 2024). *Roseburia* is a key beneficial bacterium within the gut microbiome, demonstrating anti-inflammatory and immunomodulatory properties (Faden, 2022; Huang et al., 2025). *Ruminococcus* efficiently degrades cellulose, hemicellulose, and resistant starch, playing a crucial role in stabilizing the intestinal barrier (Devendran et al., 2016). As a core component of the intestinal microbiota, *Faecalibacterium* has been shown to significantly alleviate inflammatory bowel disease, Crohn's disease, and ulcerative colitis (Xu et al., 2020, 2024). *Lactococcus* exerts its prebiotic effects through multiple mechanisms that collectively maintain intestinal microbial balance and promote host health. For instance, *Lactococcus* inhibits the proliferation of pathogens by lowering intestinal pH, producing antibiotics, and competing for adhesion sites, thus supporting host health and intestinal microbial equilibrium (Chen et al., 2021). Furthermore, *Lactococcus* has been shown to enhance intestinal barrier function and stimulate immune cell activity, thereby bolstering the body's defenses against pathogens (Boodhoo et al., 2023; Liu et al., 2024). As a key probiotic, *Bifidobacterium* possesses various physiological functions in maintaining intestinal health, regulating immunity, and nutrient metabolism, with its clinical application value widely validated. For example, *Bifidobacterium* specifically binds to intestinal epithelial cells, forming a biological barrier that prevents pathogen colonization while stimulating mucin secretion and enhancing the physical barrier function of the intestine (Dong et al., 2022; Meng et al., 2024). In livestock production, *Bifidobacterium* is extensively used as a feed additive to maintain gut microbial balance and improve host metabolism (Zhang et al., 2025). Interestingly, some of the above bacteria, including *Butyricicoccus, Christensenellaceae_R_7_group, Lactonifactor, Gordonibacter, Anaerofustis, Roseburia, Faecalibacterium, Caproiciproducens, Blautia, Roseburia, Monoglobus, Lachnospiraceae_ND3007_group, Lactococcus, Bacillus, Bifidobacterium, Ruminococcus, Intestinimonas, Coprococcus*, and *Lachnospiraceae_NK4A136_group* have also been shown to produce SCFAs. Consistent with our study, Li et al. (2023) also found that microplastics exposure can lead to a significant reduction in SCFAs-producing bacteria. Previous studies indicated that SCFAs perform significant physiological functions through various pathways. As the primary energy source for colonic epithelial cells, SCFAs not only promote the proliferation and differentiation of intestinal mucosal cells but also enhance the expression of tight junction proteins by activating the AMPK signaling pathway, thereby effectively maintaining intestinal barrier function (Ma et al., 2022). In terms of immune regulation, SCFAs inhibit the NF-κB pathway, decreasing the release of pro-inflammatory factors while simultaneously upregulating the expression of anti-inflammatory factors through a bidirectional regulatory mechanism mediated by G protein-coupled receptors (Liu et al., 2021). This characteristic suggests their potential application in the treatment of inflammatory bowel disease. Additionally, it is noteworthy that SCFAs contribute significantly to maintaining intestinal flora balance, cholesterol metabolism, and disease prevention and control (Chen et al., 2020). These functional bacteria are closely associated with host health and intestinal function. Consequently, microplastics may exacerbate gut microbial imbalance and negatively impact host health by diminishing the abundance of these beneficial bacteria.

## Conclusion

In summary, this study demonstrates that exposure to microplastics negatively impacts the antioxidant capacity and serum biochemical indicators of broiler chickens. Furthermore, microplastics exposure can lead to gut microbial dysbiosis. Given the increasing use of plastic products and the worsening issue of microplastics pollution, our research provides preliminary evidence for assessing the threat that microplastics pollution poses to broiler production. The gut microbiota is a crucial factor in maintaining host health, and its imbalance is closely associated with various diseases. Thus, future research should delve deeper into the molecular mechanisms underlying the interactions between microplastics, gut microbiota, and hosts, as well as develop personalized microecological intervention programs aimed at advancing the livestock industry and safeguarding animal health. Furthermore, future studies should also consider metabolomics analysis, SCFAs measurements, and the effects of various types and concentrations of microplastics on chicken health. However, certain limitations must be acknowledged, including the absence of experiments involving varying dosages and the relatively small sample size.

## Data Availability

The data presented in this study are publicly available. The data can be found here: https://www.ncbi.nlm.nih.gov/, accession PRJNA1328695, https://www.ncbi.nlm.nih.gov/bioproject/PRJNA1328695.
