# Supplementary material for: Microplastics: a potential threat to gut microbiota and antioxidant capacity of broiler chickens
Source: Front Microbiol. 2026 Feb 17;17:1708036. doi: 10.3389/fmicb.2026.1708036 (PMC12953438; doi:10.3389/fmicb.2026.1708036)
Supplement: Supplementary file 1 [file Table_1.docx]

**Supplementary Table S1.** Statistics of original sequence and effective sequence in the CON and MIC.

| Sample ID | Raw Reads | Clean Reads | Denoised Reads | Merged Reads | Non-chimeric Reads |
| --- | --- | --- | --- | --- | --- |
| CON1  CON2  CON3  CON4  CON5  CON6  MIC1  MIC2  MIC3  MIC4  MIC5  MIC6 | 136585  107168  136507  135836  136023  125634  97463  109773  89337  98449  97225  90105 | 120575  95251  120808  120535  120593  111968  87869  99291  81256  87875  87506  80968 | 119477  93856  119550  118972  119310  110987  87852  99247  81202  87774  87424  80680 | 109213  72585  102770  93576  107272  101468  87684  99025  80991  87663  87239  80268 | 55265  38700  50692  47995  50904  56120  72568  72935  45019  63738  64166  55595 |
